# Projected Expansion and Northwestern Shift of *Wikstroemia indica* Suitable Habitats in China Under Multiple Climate Change Scenarios: An Optimized MaxEnt Approach

**DOI:** 10.1002/ece3.72448

**Published:** 2025-11-03

**Authors:** Yangzhou Xiang, Suhang Li, Ying Liu, Qiong Yang, Jiaxin Yao, Huilin Dong, Bin Yao, Yuan Li

**Affiliations:** ^1^ School of Geography and Resources Guizhou Education University Guiyang China; ^2^ School of Biological Sciences Guizhou Education University Guiyang China; ^3^ State Key Laboratory of Tree Genetics and Breeding, Institute of Ecology Conservation and Restoration Chinese Academy of Forestry Beijing People's Republic of China; ^4^ Grasslands and Sustainable Farming, Production Systems Unit Natural Resources Institute Finland Maaninka Finland

**Keywords:** chinese medicinal herbs, climate change, maximum entropy model, species distribution, *Wikstroemia indica*

## Abstract

This study assesses the impact of climate change on the potential distribution of the traditional Chinese medicinal plant *Wikstroemia indica*, employing an optimized maximum entropy (MaxEnt) model for the first time for this species under multiple climate scenarios. Our analysis, based on 902 occurrence records and key environmental variables, provides clear evidence that climate change will significantly alter its distribution pattern. The results demonstrated that annual mean temperature (69.4% contribution) and mean diurnal temperature range (12.6% contribution) were the principal climatic factors affecting the distribution of *W. indica* . Under current climatic conditions, the total potential suitable habitat area for *W. indica* in China was calculated to be 153.31 × 10^4^ km^2^, accounting for 15.97% of China's land area. Projections indicate significant habitat expansion under future climate scenarios: under the SSP1‐2.6 scenario, the total suitable habitat area would increase by 32.0% to 202.42 × 10^4^ km^2^ by the 2090s; under the SSP5‐8.5 scenario, suitable habitat was anticipated to expand by 49.6% to 229.39 × 10^4^ km^2^. Furthermore, the distribution centroid of *W. indica* was predicted to shift 76.68–119.91 km northwestward by the 2050s. The key message is that *W. indica* demonstrates considerable resilience to climate change, with its suitable habitat expected to expand and shift northwestward. This quantitative prediction, based on robust modeling evidence, provides critical insights for future conservation planning, sustainable management, and utilization strategies for this important medicinal resource in the context of global environmental change.

## Graphical abstract

This study uses an optimized MaxEnt model to predict current and future habitat suitability of the endangered medicinal plant *Wikstroemia indica* in China under various climate scenarios. Results show a significant expansion and northwestern shift of suitable habitats, primarily influenced by annual mean temperature and diurnal temperature range, offering critical insights for conservation planning and sustainable management.

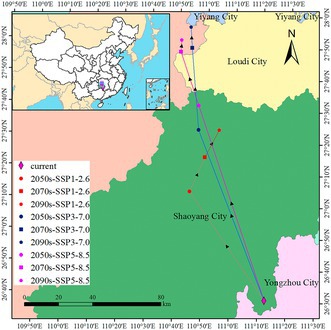

## 1 Introduction

*Wikstroemia indica* (L.) C. A. Mey., an evergreen shrub belonging to the Thymelaeaceae family and the genus *Wikstroemia*, is widely distributed across southern China, with its range encompassing provinces and regions such as the Guangxi Zhuang Autonomous Region, Guangdong Province, and Fujian Province, among other southern territories (Wu et al. 2024). For centuries, it has been valued in traditional Chinese medicine for its diverse therapeutic properties (Lee et al. 2021). The phytochemical profile of *W. indica* is characterized by a rich array of bioactive compounds, including flavonoids, alkaloids, volatile oils, and phenolic constituents (Shao et al. 2020; Shi et al. 2022; Suroowan et al. 2023; Wang, Wei, et al. 2021). These compounds confer significant pharmacological properties, leading to the development of various therapeutic formulations including tablets, capsules, granules, and tinctures for anti‐inflammatory and anti‐edematous applications (Song et al. 2024; Wu et al. 2024). Recent investigations have demonstrated promising efficacy in treating conditions such as contact hypersensitivity and breast cancer (Jegal et al. 2020; Liu et al. 2021; Yao et al. 2021; Zhang, Gao, et al. 2024).

The distribution patterns of biological species and ecosystem structures are undergoing significant alterations due to climate change, which affects the spatial configuration of suitable plant habitats (Nema et al. 2012; Zhang, Liang, et al. 2024). Rising temperatures, shifting precipitation patterns, and increased frequency of extreme weather events are progressively modifying plant habitat suitability (Newman and Noy 2023; Vystavna et al. 2021; Zeppel et al. 2014), subsequently affecting plant growth and survival trajectories, as well as broader ecosystem functions and services (Wang, Soininen, and Heino 2021). In China specifically, species distribution modeling studies have revealed that climate change is driving northward migration of suitable habitats for numerous forest plant species, while alpine plants exhibit vertical migration to higher elevations (Zhao et al. 2021). These distributional shifts potentially precipitate cascading effects on ecosystem structure and function through altered competitive dynamics, phenological desynchronization, and modified plant–pollinator interactions.

Global climate models provide essential predictive frameworks for assessing potential climate change impacts on plant habitat suitability under various emissions scenarios (Wang et al. 2022; Zhang et al. 2018). Species distribution models (SDMs), particularly MaxEnt and GARP, integrate climate projections with species occurrence records to forecast potential future suitable habitats (Horemans et al. 2024; van Steenderen and Sutton 2024). Geographic information system (GIS) technologies facilitate sophisticated spatial data processing and analysis, enabling visualization and interpretation of temporal shifts in plant habitat suitability. The integration of these technological approaches provides critical methodological frameworks for predicting and addressing climate change impacts on biodiversity (Zhang et al. 2021).

The maximum entropy (MaxEnt) model has emerged as a pivotal analytical tool in species distribution research due to its robust theoretical foundation, superior predictive accuracy, and user‐friendly interface. Employing the principle of maximum entropy, this model establishes correlative relationships between species distribution data and environmental variables to effectively predict ecological niche requirements and potential habitat distributions, thereby providing empirically grounded support for species conservation initiatives and resource management strategies (Elith et al. 2011). Despite the growing body of research utilizing MaxEnt for predicting climate change impacts on plant distributions, significant knowledge gaps remain regarding the specific responses of many medicinal plant species, including *W. indica* .

Contemporary research on *W. indica* has predominantly centered on phytochemical characterization and pharmacological efficacy evaluation (Keem et al. 2024; Lu et al. 2024). However, there exists a notable deficiency in investigations exploring the potential distributional responses of this plant to climate change scenarios. This research lacuna is particularly concerning given the ecological and economic importance of *W. indica* , as climate‐induced distributional shifts could significantly impact both wild populations and cultivation prospects. Understanding potential range shifts is therefore essential for developing effective conservation strategies and ensuring sustainable utilization of this valuable medicinal resource.

In this research, we applied an optimized MaxEnt model in conjunction with comprehensive geographical distribution data and environmental variables to predict future distribution patterns of suitable *W. indica* habitats across China. This investigation aimed to provide empirical support for conservation planning and sustainable resource management of this species, while also offering methodological insights applicable to distribution studies of other medicinally important plant species. The specific objectives of this study were to: (1) employ the ENMeval package to optimize the MaxEnt model for high‐precision prediction of *W. indica* distribution; (2) identify and quantify the impact of key environmental factors influencing the distribution of *W. indica* ; and (3) simulate the spatiotemporal dynamics of *W. indica* distribution and potential migration patterns under current and projected climate change scenarios. These findings would contribute to the development of ecologically informed adaptive conservation strategies, supporting biodiversity preservation and sustainable development objectives in the context of global environmental change.

Despite the pharmacological importance of *W. indica* , it remains unclear how its geographic distribution in China will respond to future climate change. This study aims to address this critical gap by answering the following scientific question: How will multiple climate change scenarios alter the extent, spatial pattern, and centroid location of suitable habitats for *W. indica* in China? By integrating an optimized MaxEnt model with CMIP6 projections, we seek not only to predict range shifts but also to identify the key climatic drivers governing these changes, thereby providing a scientific basis for the conservation and sustainable use of this medicinal species under global change.

## 2 Materials and Methods

### 2.1 Acquisition and Processing of *W. indica* Distribution Data

The geographical distribution information of *W. indica* in China was compiled from multiple authoritative botanical databases: the Chinese Virtual Herbarium (CVH, https://www.cvh.ac.cn, accessed on July 10, 2024), the Global Biodiversity Information Facility (GBIF, https://www.gbif.org, accessed on July 16, 2024), the National Specimen Information Infrastructure of China (NSII, http://www.nsii.org.cn/2017/home.php, accessed on July 6, 2024), and the China National Knowledge Infrastructure (CNKI, https://www.cnki.net, accessed on July 8, 2024). To develop distribution points with explicit coordinates, longitude and latitude data were extracted from the mentioned databases. Where precise geographic coordinates were lacking, provincial, county, township, or village‐level collection details, were used in the online georeferencing platform (http://jingweidu.757dy.com/) to spatially reference the locality descriptions. Through this comprehensive approach, a total of 902 non‐duplicate occurrence records for *W. indica* in China were collected.

**FIGURE 1:**
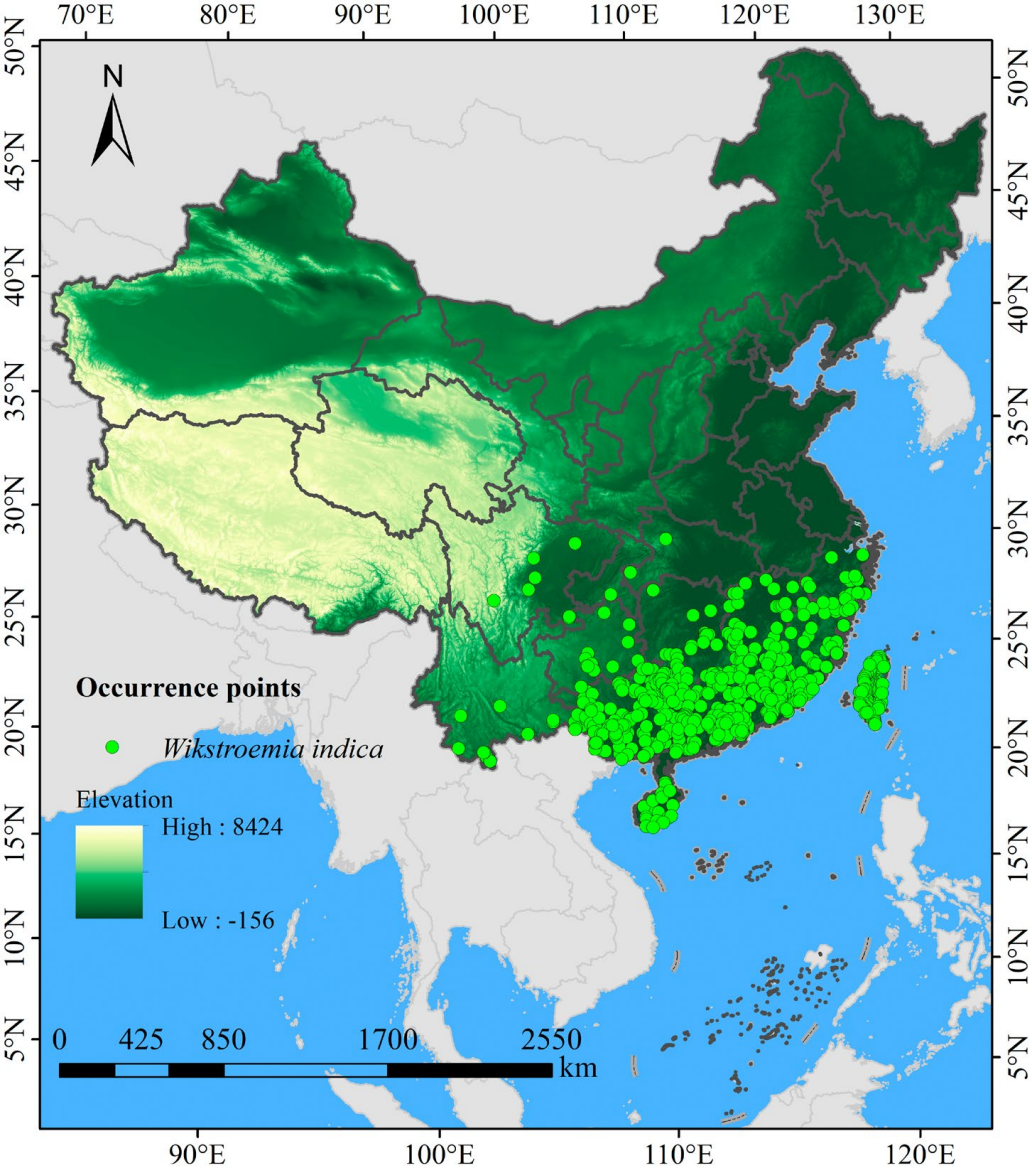
Distribution of occurrence records of *W. indica* in China.

To mitigate spatial autocorrelation and reduce model overfitting risk, spatial filtering was implemented following established protocols (Whitford et al. 2024). A grid layer with a resolution of 2.5′ × 2.5′ was loaded in ArcGIS 10.8 to visualize and optimize the distribution point selection for *W. indica* . Using spatial analysis tools, the Euclidean distance from each record point to the center of its corresponding grid cell was calculated. Subsequently, only the single occurrence record closest to each grid center was retained, ensuring a spatially balanced distribution of occurrence points. This spatial filtering process yielded 624 valid distribution points (Figure 1), providing a robust dataset for model calibration while minimizing the potential for spatial sampling bias to influence model predictions. Spatial analyses were performed using China's official base map (Approval No. GS (2023)2762) at a 1:10,000,000 scale, which was converted to shapefile format for GIS processing.

### 2.2 Acquisition and Processing of Environmental Parameters

Nineteen bioclimatic variables (bio1‐bio19) representing contemporary climate conditions and future climate scenarios were obtained from WorldClim (https://www.worldclim.org, accessed on May 18, 2024), supplemented with SRTM elevation data at 2.5 arc‐minute resolution. Topographic derivatives including slope aspect and gradient were generated from the elevation data using ArcGIS 10.8 spatial analyst tools. All environmental datasets were standardized to a common 2.5‐min spatial resolution and converted from tif format to asc format to meet the input requirements of MaxEnt 3.4.4 software (https://biodiversityinformatics.amnh.org/open_source/maxent/, accessed on August 2, 2024).

**FIGURE 2:**
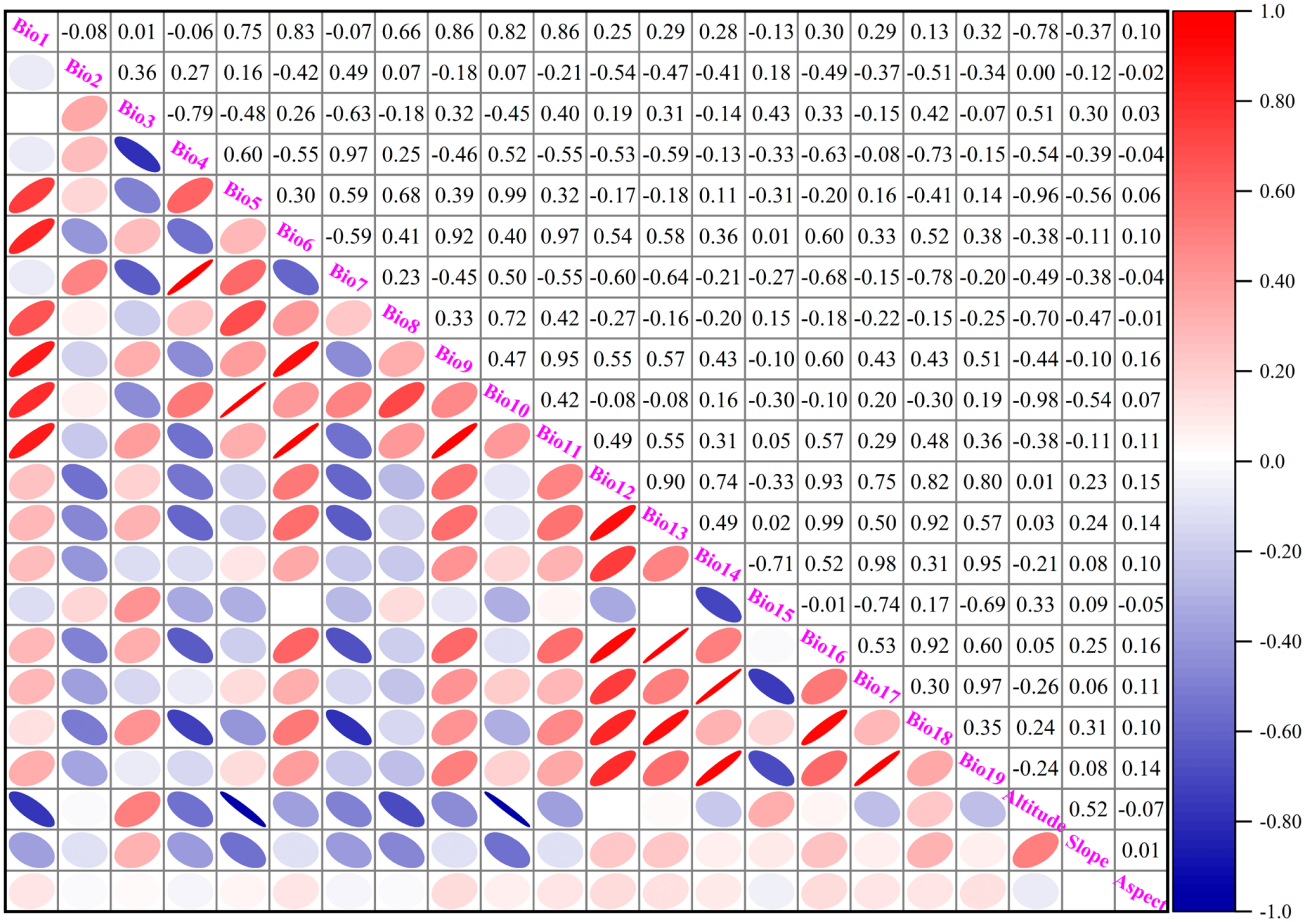
Correlation relationship of 22 environmental variables for *W. indica* .

Multicollinearity among environmental variables can compromise the accuracy and interpretability of species distribution predictions (Chen et al. 2024). To address this issue, Pearson correlation analysis was conducted on 22 environmental variables (19 climatic factors and 3 topographic factors) using IBM SPSS Statistics software (version 26) to obtain correlation coefficients (Table 1). The contribution rates of the 22 environmental variables to the distribution of *W. indica* were calculated using MaxEnt 3.4.4 software (Figure 2). To improve the predictive accuracy of the MaxEnt model and reduce redundant information, variables with an absolute correlation coefficient below 0.75 were retained (Yang, Xiang, et al. 2024). When the correlation coefficient |R| between two environmental variables was greater than 0.75, the factor with the smaller contribution rate was eliminated. This systematic variable selection process resulted in the retention of nine key environmental predictors: Bio1 (Annual mean temperature), Bio2 (Mean diurnal temperature range), Bio5 (Max temperature of warmest month), Bio7 (Temperature annual range), Bio12 (Annual precipitation), Bio15 (Precipitation seasonality), Bio19 (Precipitation of coldest quarter), Aspect, and Slope.

**TABLE 1:** Twenty‐two environmental factors used in this study.

Variables	Description	Units	Range	Contribution rate (%)
Bio1	Annual mean temperature	°C	8.8–25.8	0.3
Bio2	Mean diurnal range (Mean of monthly)	°C	4.9–13.1	0.8
Bio3	Isothermality (Bio2/Bio7) (×100)		21.3–53.6	1.5
Bio4	Standard deviation of temperature seasonality		284.4–893.7	1.7
Bio5	Max temperature of warmest month	°C	20.3–34.3	3.6
Bio6	Min temperature of coldest month	°C	−7.3‐17.6	3.8
Bio7	Temperature annual range (Bio5‐Bio6)	°C	13.2–32.8	0.3
Bio8	Mean temperature of wettest quarter	°C	14.9–28.9	2.3
Bio9	Mean temperature of driest quarter	°C	1.8–22.8	0.6
Bio10	Mean temperature of warmest quarter	°C	14.9–28.9	0.9
Bio11	Mean temperature of coldest quarter	°C	1.8–21.9	17.9
Bio12	Annual precipitation	mm	876.0–4256.0	0.6
Bio13	Precipitation of wettest month	mm	167.0–1064.0	0.1
Bio14	Precipitation of driest month	mm	2.0–196.0	0.4
Bio15	Variation of precipitation seasonality		23.4–109.8	0.5
Bio16	Precipitation of wettest quarter	mm	432.0–2661.0	27.5
Bio17	Precipitation of driest quarter	mm	9.0–625.0	0.6
Bio18	Precipitation of warmest quarter	mm	347.0–2661.0	30.2
Bio19	Precipitation of coldest quarter	mm	9.0–635.0	0.1
Altitude	Altitude	m	0.0–3120.0	0.2
Aspect	Aspect	°	0.6–359.6	0.9
Slope	Slope	°	0.0–7.7	0.3

To assess potential distributional responses to future climate conditions, bioclimatic variable projections from the BCC‐CSM2‐MR model were selected from the Coupled Model Intercomparison Project Phase 6 (CMIP6). This model was chosen based on its demonstrated reliability in simulating climate conditions across China, particularly its superior performance in representing regional temperature and precipitation patterns compared to other CMIP6 models (Zhong et al. 2023). The spatial resolution of the future climate data matched the contemporary data at 2.5′ × 2.5′. Three time periods were considered for projections: 2050s (2041–2060), 2070s (2061–2080), and 2090s (2081–2100). To capture a range of potential climate futures, three Shared Socioeconomic Pathway (SSP) scenarios were selected: SSP1‐2.6 (sustainable development with low emissions), SSP3‐7.0 (regional rivalry with high emissions), and SSP5‐8.5 (fossil‐fueled development with very high emissions), representing forcing levels of 2.6, 7.0, and 8.5 W m^−2^ by 2100, respectively (He et al. 2023).

### 2.3 MaxEnt Model Optimization and Modeling

#### 2.3.1 Optimization of MaxEnt Model

Overfitting in uncalibrated models can skew predictions, potentially leading to ineffective conservation strategies for *W. indica* (Kong et al. 2019; Velazco et al. 2020). Thus, fine‐tuning MaxEnt model parameters is crucial for accurately assessing climate change impacts on its future distribution. ENMeval was chosen for its specialized MaxEnt framework, providing rigorous, reproducible parameter tuning to enhance ecological realism over default or generalized approaches. The “ENMeval” R package was employed to optimize the MaxEnt model by adjusting key parameters: the regularization multiplier (RM) and feature combination (FC) (Warren et al. 2021). To assess the forecasting accuracy of the MaxEnt model on *W. indica* , the 624 occurrence records were randomly split into a training set (75%) and a testing set (25%) employing k‐fold cross‐validation. The model's responsiveness to regularization was examined by setting eight RM values from 0.5 to 4.0, each increased by 0.5 (Shi et al. 2024). The configuration of the MaxEnt model allowed for the automatic selection from hinge features (H), linear features (L), interaction features (P), quadratic features (Q), and threshold features (T). Nine FC parameters, namely H, HPT, L, LQ, LQH, LQHP, LQHPT, QHP, QHPT, were defined. The model fit and complexity were evaluated using the Akaike Information Criterion Correction (delta.AICc). To reduce the risk of overfitting, consideration was also given to the discrepancy in Area Under the Curve (AUC) scores between the training and validation subsets (AUC.DIFF). The parameter set that exhibited the smallest increase in delta.AICc was selected as the optimal configuration for the model (Pearson et al. 2007).

#### 2.3.2 MaxEnt Model Parameter Setting

For distribution modeling of *W. indica* , the 624 georeferenced occurrence points and nine key environmental variables were input into MaxEnt 3.4.4. Following optimization results, the RM was set to 0.5 and FC to LQ (linear and quadratic features). The model was configured to allocate 25% of occurrence data to testing and 75% to training. Cross‐validation was implemented using a subsample approach with 10 replications, and model outputs were formatted as logistic values. All other parameters were maintained at default settings. The final predictive distribution map, representing the average of ten model iterations, was generated in ASCII format. The relative contribution of each environmental variable to the model was quantified to identify the primary determinants of *W. indica* habitat suitability.

#### 2.3.3 Evaluation of MaxEnt Model Results

The predictive performance of the MaxEnt model for *W. indica* distribution was evaluated using the area under the receiver operating characteristic curve (AUC). This metric directly quantifies model discrimination ability, with AUC values between 0.9 and 1.0 indicating excellent predictive performance; 0.8–0.9 suggesting good performance; 0.7–0.8 indicating fair performance; 0.6–0.7 suggesting poor performance; and 0.5–0.6 indicating model prediction failure (Zhao et al. 2021). The AUC metric is particularly valuable as it provides a threshold‐independent assessment of model performance across the entire range of possible presence‐absence classification thresholds.

### 2.4 The Suitable Area Division of *W. indica*

The output generated by the MaxEnt 3.4.4 model in asc format was converted into tif format raster data using ArcGIS 10.8 software. To delineate the potential suitable habitats for *W. indica* with refinement, this study employed the natural breaks method and, in conjunction with the species' geographical distribution characteristics, categorized the study area into four suitability grades: unsuitable (0–0.1), low suitability (0.1–0.3), moderate suitability (0.3–0.5), and high suitability (0.5–1) (Yang, Xiang, et al. 2024).

### 2.5 Spatial Distribution Pattern of Suitable Areas of *W. indica*

A multi‐temporal suitable habitat prediction pattern was established in this study, categorizing habitat changes into retention, loss, and gain. Following the method of Xu, Ye, et al. (2023), a logical threshold of 0.1 was set based on the suitable habitat prediction data for *W. indica* to delineate suitable from unsuitable habitats, ensuring the coverage of various suitability grades. Subsequently, a matrix was constructed to reflect the changes in suitable habitats from past to present and into future climate scenarios, analyzing the dynamic changes in habitats. The value changes in the matrix (0 to 1 indicating gain, 1 to 0 indicating loss, and 1 remaining unchanged indicating retention) revealed the transformation of habitats. Finally, the matrix values were transformed into attribute data using ArcGIS 10.8, achieving the visualization of the spatial changes in the suitable habitats for *W. indica* .

### 2.6 Centroid Migration of *W. indica* Geographic Distribution

The distribution of *W. indica* for three future periods and under three climate scenarios was initially predicted using the Maxent 3.4.4 model, and the average of ten repetitions was calculated to generate ASC files. Subsequently, these files underwent a 3D reclassification process, with a threshold set to select habitats where the species' presence probability was greater than 0.1. This thresholding was applied to identify the suitable habitats for *W. indica* . Subsequently, the “mean Center” tool in ArcGIS 10.8 software was utilized to convert the suitable area's tif data into point data, extracting the central points of *W. indica* distribution. By repeating this process, the distribution centers for the three periods and three climate scenarios were obtained and merged into a single vector dataset. Finally, the “points to line” tool was used to connect the centroids of different periods under the same SSP scenario, thereby plotting the centroid migration map of the suitable habitats for *W. indica* over time.

## 3 Results

### 3.1 Enhancement and Assessments of Maximum Entropy Model

**FIGURE 3:**
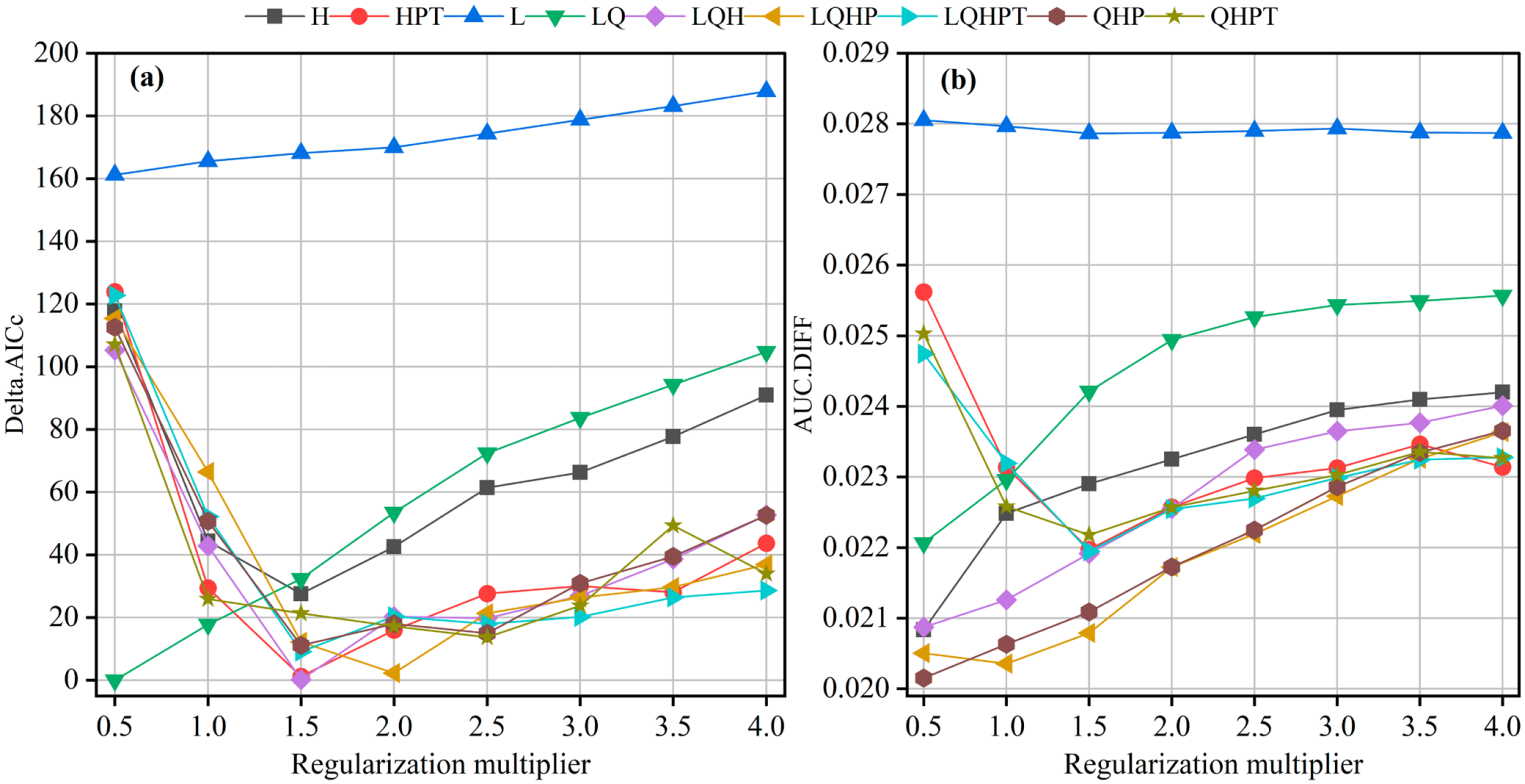
*W. indica* 's (a) Delta.AICc and (b) AUC.DIFF generated by ENMeval. The legends represent various feature categories (H, Hinge features; L, Linear features; P, Product features; Q, Quadratic features; T, Threshold features).

Parameter optimization of the MaxEnt model through the ENMeval package suggested that configuring the RM to 0.5 and selecting a FC of linear and quadratic (LQ) features yielded the lowest delta.AICc value, resulting in an optimal AICc score of zero (Figure 3a). Subsequent evaluation indicated that this optimized model configuration exhibited an 8.40% improvement in AUC.DIFF compared to the default parameterization (RM = 1.0 and FC = LQHP) (Figure 3b). Consequently, the final MaxEnt model parameters for predicting the potential geographical distribution of *W. indica* under climate change scenarios were established as RM = 0.5 and FC = LQ.

**FIGURE 4:**
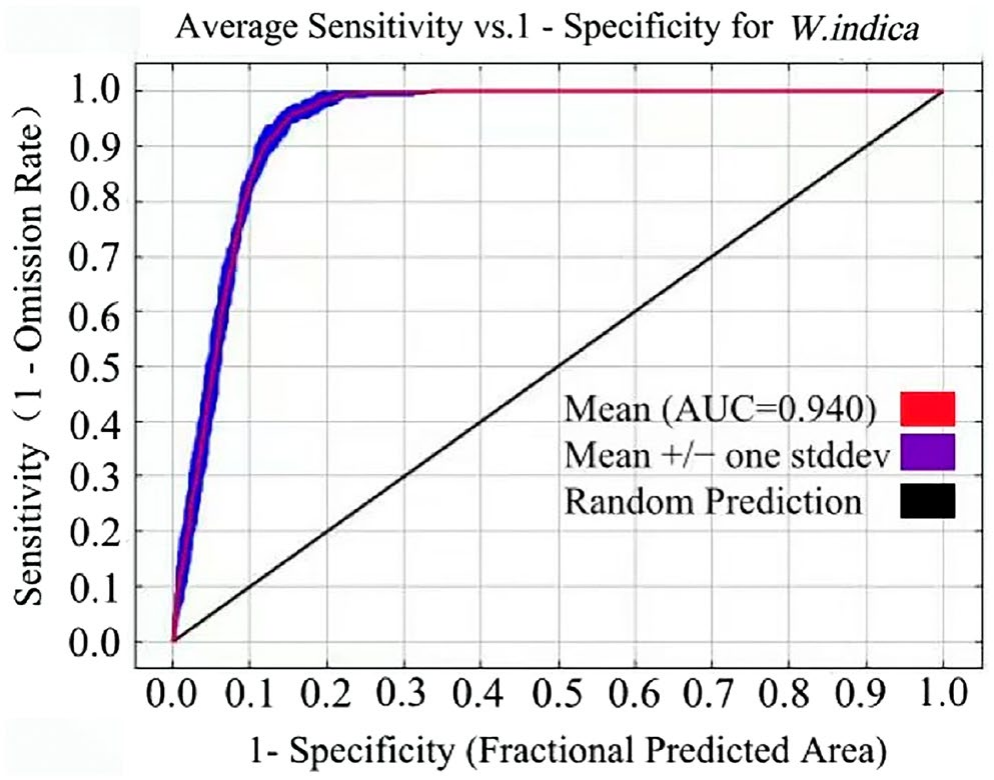
ROC curve of distribution prediction of *W. indica*.

The optimized MaxEnt model was executed independently ten times to ensure statistical robustness, yielding an average AUC value of 0.940 (Figure 4). This AUC value substantially exceeds the 0.9 threshold for excellent model performance, confirming the high predictive accuracy of the model. The same optimized parameter configuration was applied to predict the potential distribution of *W. indica* under three future time periods and three SSP scenarios, maintaining consistent AUC values of 0.940 across all projections. These results demonstrate the model's reliability for predicting the current and future potential distribution of *W. indica* throughout China under various climate change scenarios.

### 3.2 Primary Environmental Drivers and Their Response Curves

**FIGURE 5:**
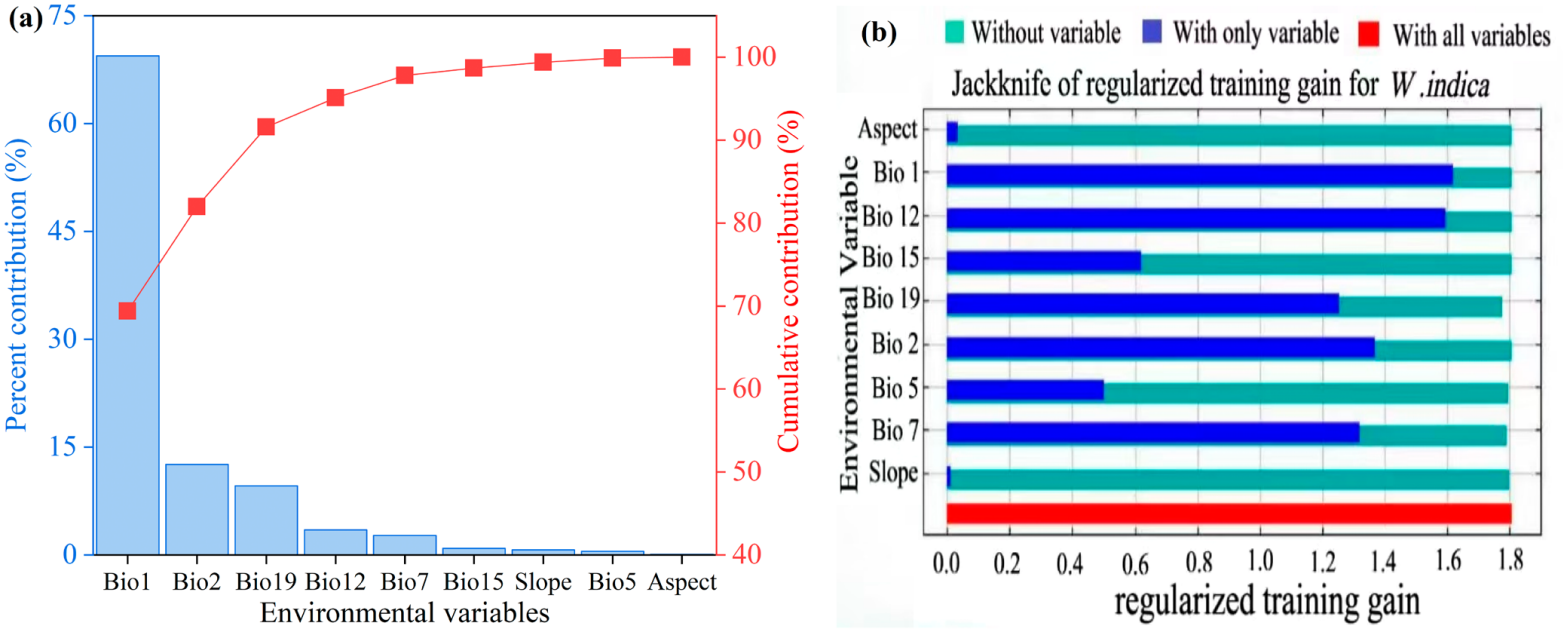
Contribution (a) and Jackknife test (b) of the environmental variables.

In this study, the output results from the optimized MaxEnt model were utilized to compare the importance of variables using the jackknife method (Figure 5a) and to reveal the main environmental factors affecting the distribution of *W. indica* through normalized training gain (Figure 5b). From the contribution rates (Figure 5a), it was found that Annual mean temperature (Bio1) had the highest contribution rate to the model predictions, at 69.4%; followed by Mean diurnal range (Mean of monthly) (Bio2) with a contribution rate of 12.6%; Precipitation of coldest quarter (Bio19), Annual precipitation (Bio12), and Temperature annual range (Bio5‐Bio6) (Bio7) ranked third, fourth, and fifth, with contribution rates of 9.6%, 3.5%, and 2.7%, respectively. The cumulative contribution rate of the top five factors was 97.8%.

The cumulative contribution rate of the seven climatic factors was 99.2%, while that of the two topographic factors was only 0.8%. In terms of the Jackknife test (Figure 5b), the top five environmental factors are Annual mean temperature (Bio1), Annual precipitation (Bio12), Mean diurnal range (Mean of monthly) (Bio2), Temperature annual range (Bio5‐Bio6) (Bio7), and Precipitation of the coldest quarter (Bio19). Accordingly, Annual mean temperature (Bio1), Mean diurnal range (Mean of monthly) (Bio2), Precipitation of the coldest quarter (Bio19), and Annual precipitation (Bio12) are the dominant environmental factors influencing the geographical distribution pattern of *W. indica* .

**FIGURE 6:**
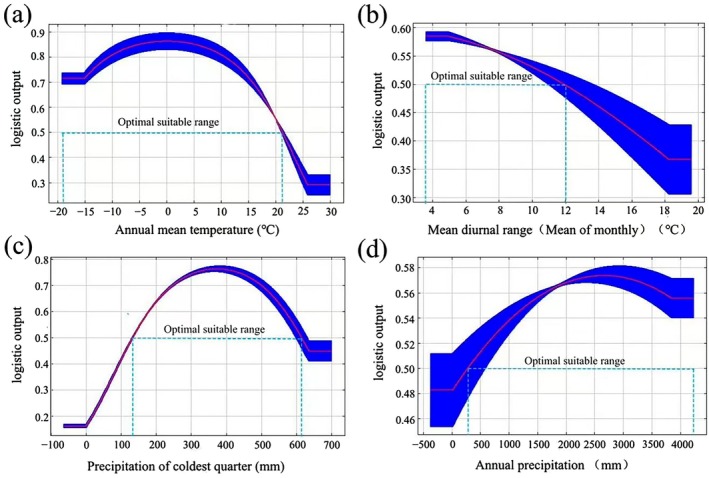
Response curves of the effect of major environmental factors on occurrence probability of *W. indica*. (a) Response curves of Annual mean temperature; (b) Response curves of Mean diurnal range; (c) Response curves of Precipitation of coldest quarter; (d) Response curves of Annual precipitation. The gap between the two parallel dashed lines denotes the ideal environmental parameter scope.

Climate response curves were developed to ascertain the optimal threshold range for the principal environmental factors that influence the geographic distribution of *W. indica* . The spatial units with a probability value *p* ≥ 0.5 were designated as the most suitable distribution areas (Yang, Xiang, et al. 2024). The highest distribution probability for *W. indica* was observed under the following climatic conditions: annual mean temperature (Bio1) ranging from −18.9°C to 21.3°C, mean diurnal range (Bio2) between 3.6 and 11.9, precipitation of coldest quarter (Bio19) from 131.8 to 613.2 mm, and annual precipitation (Bio12) between 289.4 and 4210.7 mm (Figure 6).

### 3.3 Current Potential Suitable Areas of *W. indica* in China

**FIGURE 7:**
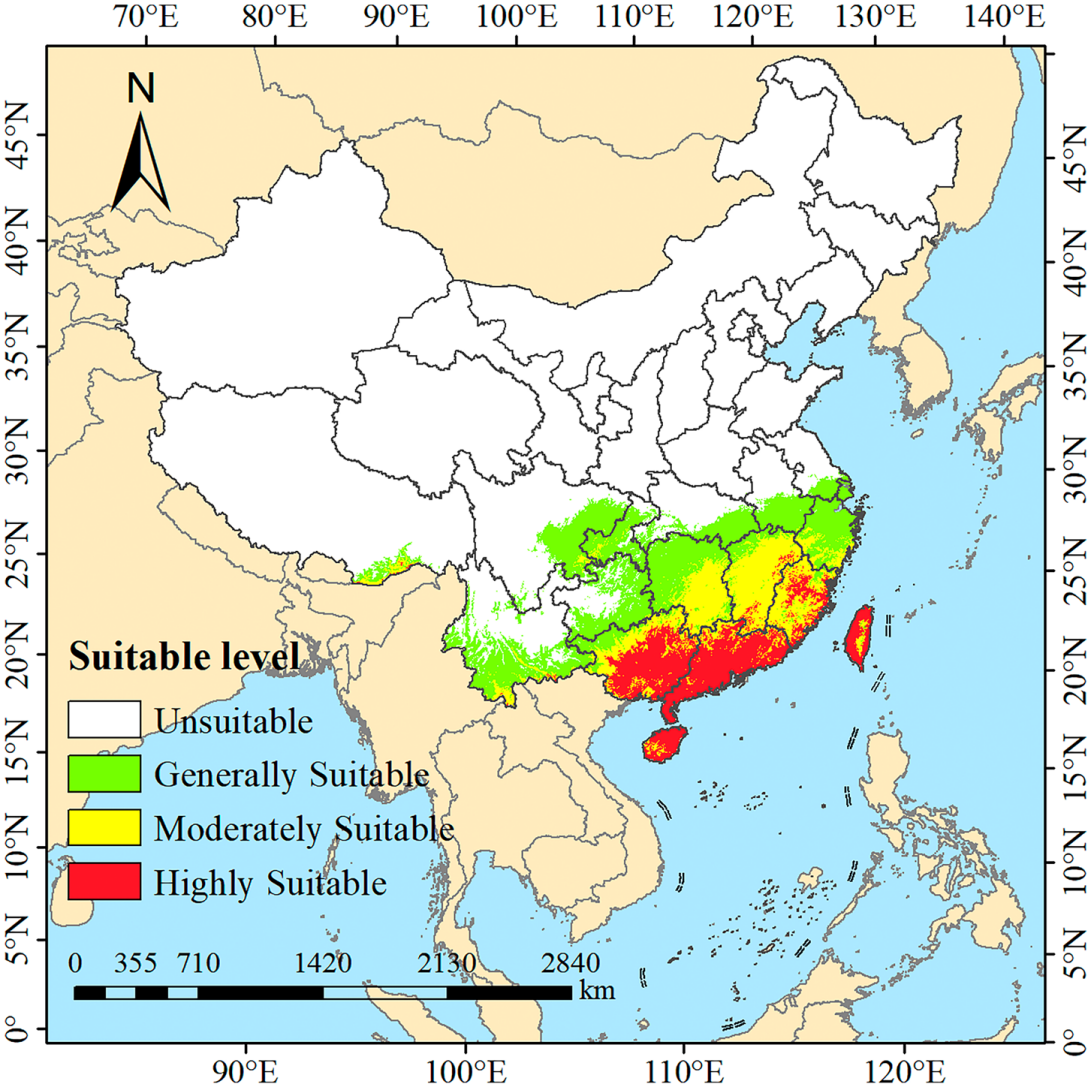
Potential distribution of *W. indica* in China under current climate.

Under the current climatic conditions, the potential total suitable habitat area for *W. indica* is 153.31 × 10^4^ km^2^, accounting for 15.97% of China's land area (Figure 7). The low suitability area for *W. indica* is approximately 78.45 × 10^4^ km^2^, representing 8.17% of China's land area, which mainly includes regions such as Chongqing Municipality, Guizhou Province, Zhejiang Province, the southern part of Yunnan Province, the eastern part of Sichuan Province, the western part of Hunan Province, the eastern part of Hubei Province, the southern part of Anhui Province, the southern part of Jiangsu Province, and the southern part of the Tibet Autonomous Region. The moderately suitable area is about 41.97 × 10^4^ km^2^, constituting 4.37% of China's land area, primarily encompassing areas such as Jiangxi Province, the northern part of Guangxi Province, the southern part of Hunan Province, and the western part of Fujian Province. The high suitability area is roughly 32.89 × 10^4^ km^2^, making up 3.43% of China's land area, and is mainly located in Guangxi Province, Guangdong Province, Hainan Province, Taiwan Province, and some parts of Fujian Province. The nonsuitable areas for *W. indica* primarily cover the northwestern and northern regions of China.

### 3.4 Suitable Areas of *W. indica* Under Future Climate Change

**FIGURE 8:**
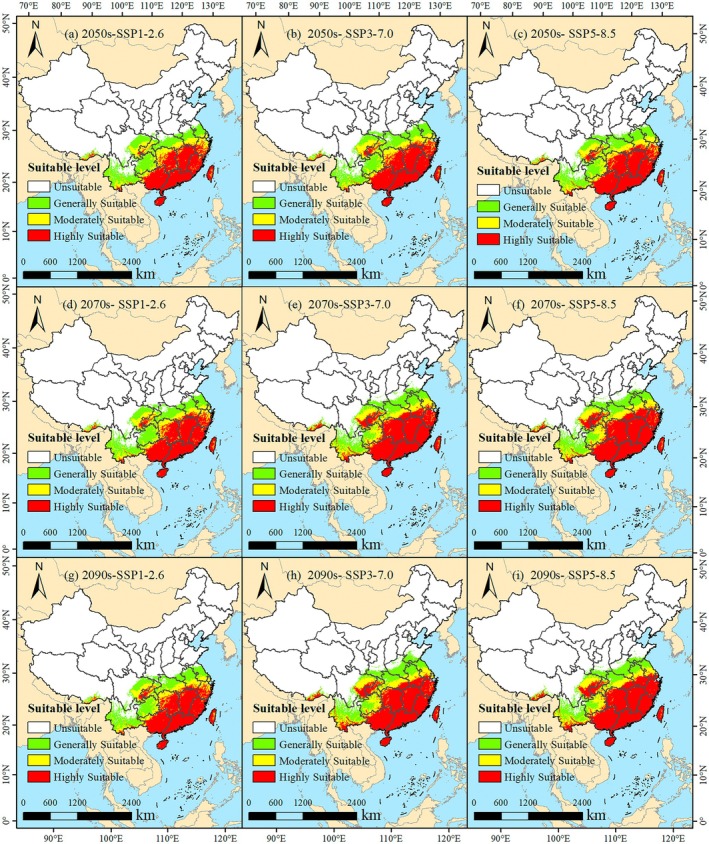
Suitable area of *W. indica* under future climate scenarios. (a) 2050s‐SSP1‐2.6; (b) 2050s‐SSP3‐7.0; (c) 2050s‐SSP5‐8.5; (d) 2070s‐SSP1‐2.6; (e) 2070s‐SSP3‐7.0; (f) 2070s‐SSP5‐8.5; (g) 2090s‐SSP1‐2.6; (h) 2090s‐SSP3‐7.0; (i) 2090s‐SSP5‐8.5.

Under the SSP1‐2.6 scenario, the total suitable habitat for *W. indica* in the 2050s is projected to be 191.30 × 10^4^ km^2^. The highly suitable area constitutes the largest portion of this total, reaching 82.32 × 10^4^ km^2^. A continuing trend is observed into the 2090s, with the total suitable area increasing slightly to 202.42 × 10^4^ km^2^ (Table 2). The spatial distribution shows that the high suitability areas are primarily located along the coastal regions from Guangdong Province to the southern part of Zhejiang Province, as well as in the central part of Hunan Province (Figure 8a,g).

**TABLE 2:** Potential suitable area of *W. indica* in different periods (unit: 10^4^ km^2^).

Period	Unsuitable area	TSA	GSA	MSA	HSA
Current	806.69	153.31	78.45	41.97	32.89
2050s‐SSP1‐2.6	768.70	191.30	64.19	44.79	82.32
2070s‐SSP1‐2.6	762.35	197.65	65.31	42.08	90.26
2090s‐SSP1‐2.6	757.58	202.42	65.53	45.86	91.03
2050s‐SSP3‐7.0	756.78	203.22	67.53	43.56	92.13
2070s‐SSP3‐7.0	733.93	226.07	62.96	46.04	117.07
2090s‐SSP3‐7.0	728.65	231.35	64.47	47.79	119.09
2050s‐SSP5‐8.5	749.81	210.19	66.75	46.82	96.62
2070s‐SSP5‐8.5	733.18	226.82	60.87	46.54	119.41
2090s‐SSP5‐8.5	730.61	229.39	59.69	45.59	124.11

Abbreviations: GSA, generally suitable area; HAS, highly suitable area; MSA, moderately suitable area; TSA, total suitable area.

Under the SSP3‐7.0 scenario, the forecast for the 2050s indicates a total suitable habitat area of 203.22 × 10^4^ km^2^, with the high suitability area being 92.13 × 10^4^ km^2^ (Table 2). By the 2090s, the total suitable area expands further to 231.35 × 10^4^ km^2^, and the high suitability area is also observed to increase in extent (Figure 8b,h). The geographical distribution under this scenario shows expansion, particularly in the southern part of Yunnan Province and the southeastern part of the Tibet Autonomous Region.

Under the SSP5‐8.5 scenario, the estimated total suitable area for the 2050s is 210.19 × 10^4^ km^2^, with a high suitability area of 96.62 × 10^4^ km^2^ (Table 2). By the 2090s, the total suitable area reaches 229.39 × 10^4^ km^2^. The high suitability area demonstrates significant growth during this period, with its northern boundary extending to include the southern part of Jiangsu Province (Figure 8c,i). The predictions across all scenarios consistently show an expansion in both the total suitable habitat area and the distribution range for *W. indica* from the 2050s to the 2090s.

### 3.5 Variation Characteristics of Suitable Area of *W. indica* Under Future Climate Scenarios

**FIGURE 9:**
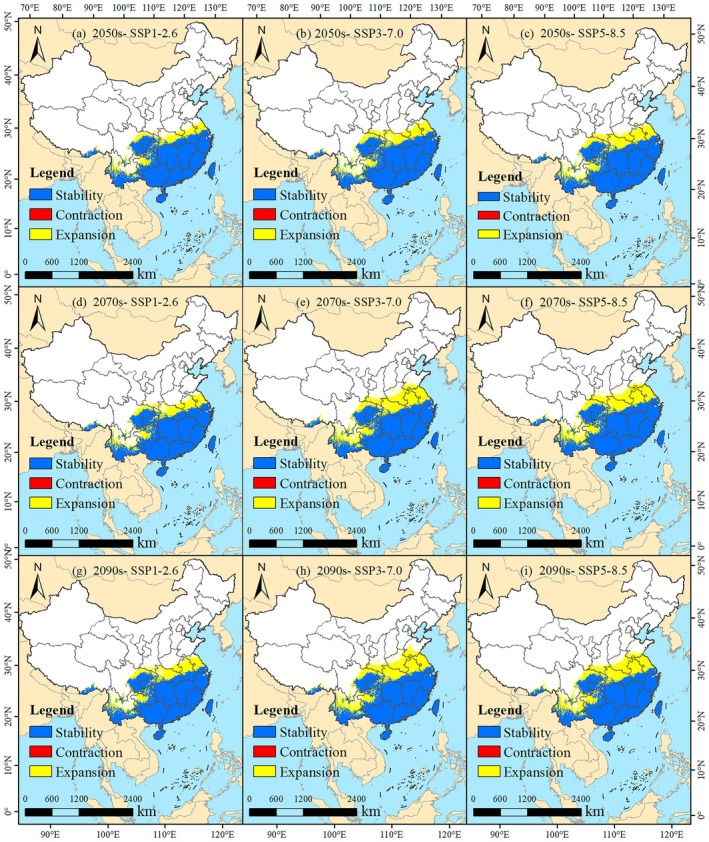
Changes in the habitat area of *W. indica* under different climate scenarios. (a) 2050s‐SSP1‐2.6; (b) 2050s‐SSP3‐7.0; (c) 2050s‐SSP5‐8.5; (d) 2070s‐SSP1‐2.6; (e) 2070s‐SSP3‐7.0; (f) 2070s‐SSP5‐8.5; (g) 2090s‐SSP1‐2.6; (h) 2090s‐SSP3‐7.0; (i) 2090s‐SSP5‐8.5.

Under the SSP1‐2.6 scenario, the retained suitable habitat area for *W. indica* in the 2050s is 187.13 × 10^4^ km^2^, with a retention rate of 80.08%. The habitat loss is minimal (0.04 × 10^4^ km^2^; 0.02%), while expansion is considerable (46.51 × 10^4^ km^2^; 19.90%; Figure 9a). By the 2070s, the retained area remains stable, though the retention rate decreases to 77.54%. The expanded area grows to 54.17 × 10^4^ km^2^, with an expansion rate of 22.45% (Figure 9d). In the 2090s, the retained area slightly declines to 187.12 × 10^4^ km^2^ (retention rate: 75.69%), while expansion continues to increase, reaching 60.05 × 10^4^ km^2^ (24.29%; Figure 9g). Overall, under this low‐emission scenario, habitat retention remains largely stable with negligible loss, while expansion increases consistently.

Under the SSP3‐7.0 scenario, the retained area in the 2050s is also 187.13 × 10^4^ km^2^, but the retention rate is lower (75.39%). Loss remains minimal (0.04 × 10^4^ km^2^; 0.01%), while expansion is higher than under SSP1‐2.6 (61.04 × 10^4^ km^2^; 24.59%; Figure 9b). By the 2070s, the retained area increases slightly to 187.15 × 10^4^ km^2^, though the retention rate drops to 67.81%. Expansion rises significantly to 88.84 × 10^4^ km^2^ (32.19%; Figure 9e). In the 2090s, the retained area remains unchanged, with a further decline in retention rate to 66.25%, while expansion increases to 95.32 × 10^4^ km^2^ (33.74%; Figure 9h). This scenario shows a slight increase in retained area, minimal loss, and a strong upward trend in expansion.

Under the SSP5‐8.5 scenario, the retained area in the 2050s is 187.14 × 10^4^ km^2^, with a retention rate of 72.89%. Habitat loss is very low (0.02 × 10^4^ km^2^; 0.01%), while expansion is 69.57 × 10^4^ km^2^ (27.10%; Figure 9c). By the 2070s, the retained area increases to 187.15 × 10^4^ km^2^, though the retention rate falls to 67.57%. Expansion rises to 89.82 × 10^4^ km^2^ (32.43%; Figure 9f). In the 2090s, retention remains steady, with the retention rate at 66.83%, and expansion reaches 92.87 × 10^4^ km^2^ (33.16%; Figure 9i). Under this high‐emission pathway, the retained area shows a slight increasing trend, loss remains negligible, and expansion increases markedly over time.

### 3.6 Centroid Migration of *W. indica* Under Different Climatic Conditions

**FIGURE 10:**
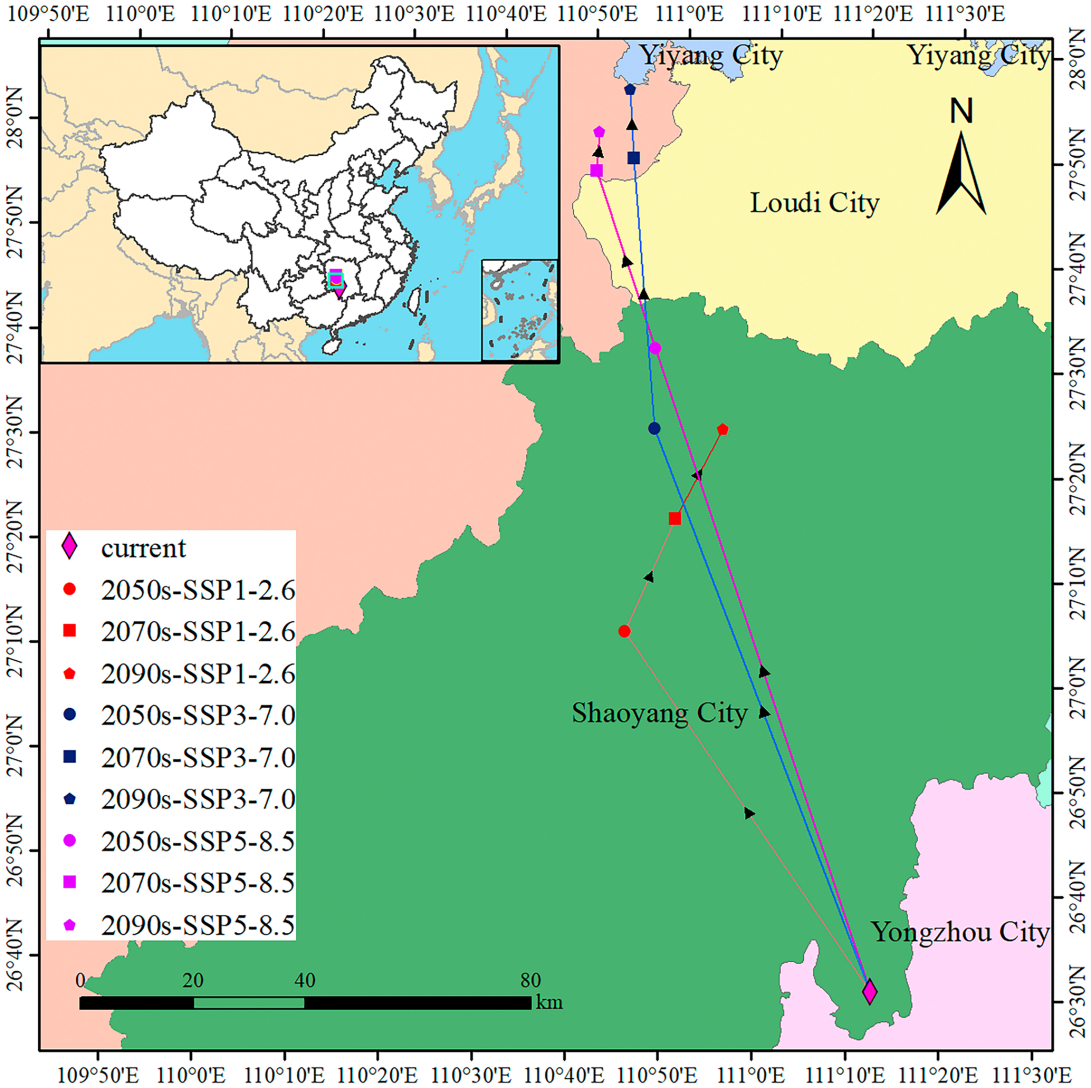
Shifts in the geographic center of *W. indica* habitats across climate scenarios.

The centroid of the potentially suitable distribution area for *W. indica* in China demonstrates a consistent northwestward shift under future climate scenarios (Figure 10). The current centroid is located in Jingwei Township, Xinxing County, Hunan Province (111°13′ E, 26°32′ N). Under the SSP1‐2.6 scenario, the centroid is projected to migrate 76.68 km northwest to Nanyue Miao Town, Longhui County (110°49′ E, 27°8′ N) by the 2050s. A further shift of 22.32 km to Liudu Zhai Town, Longhui County (110°55′ E, 27°18′ N) is expected in the 2070s, followed by an 18.43 km movement to Qijiang Town, Longhui County (111°1′ E, 27°27′ N) by the 2090s. Under the SSP3‐7.0 scenario, a more pronounced northwestward migration of 106.34 km to Siqianmen Town, Longhui County (110°54′ E, 27°27′ N) is anticipated in the 2050s. The centroid is then projected to move 47.81 km to Fengjia Town, Xupu County (110°53′ E, 27°53′ N) in the 2070s, and another 13.34 km to Jinlan Village, Xupu County (110°53′ E, 27°60′ N) by the 2090s. Under the SSP5‐8.5 scenario, the centroid is expected to shift 119.91 km northeast to Yatian Town, Longhui County (110°54′ E, 27°35′ N) in the 2050s. This will be followed by a 33.19 km migration to Youyang Township, Xupu County (110°49′ E, 27°52′ N) in the 2070s, and a final movement of 6.74 km to Jiangdong Village, Xupu County (110°50′ E, 27°56′ N) in the 2090s.

## 4 Discussion

### 4.1 Model Parameter Optimization and Reliability of Prediction Results

Ecological niche models are employed to estimate species' requirements using specific algorithms, aimed at predicting the potential distribution areas of species. Therefore, enhancing the accuracy of model predictions is crucial in the construction of ecological niche models. The ENMeval package, designed specifically for optimizing parameters of the Maxent model, selects the optimal modeling parameters based on predictive accuracy and model complexity (Kass et al. 2021; Muscarella et al. 2014). Compared to other similar R packages, the ENMeval package employs a more rigorous model evaluation process and is capable of automatically selecting the best model. Additionally, it supports testing different combinations of environmental variables, and compared to earlier ecological niche models, it is more intelligent, capable of integrating various extrapolation options to determine the final model. These features of ENMeval facilitate the generation of more accurate and robust ecological niche models by Maxent.

In this study, the default settings of the MaxEnt model were RM = 1, FC = LQHP, and delta.AICc = 66.44. After applying the ENMeval software package for parameter optimization using the distribution point data of the species and its related environmental factors, the parameters of the MaxEnt model were adjusted to RM = 0.5, FC = LQ, and delta.AICc was reduced to 0. This optimization significantly improved the balance between model fit and complexity, thereby enhancing the reliability of model predictions. Previous studies have confirmed that the Maxent model optimized with ENMeval has a high degree of accuracy in predicting the distribution of tree species such as *Liriodendron chinense* (Hemsl.) Sarg., *Cunninghamia lanceolata* (Lamb.) Hook., and *Keteleeria davidiana* (Pinaceae) (Bai et al. 2024; Zhang et al. 2023; Zhao et al. 2021). When this study used the MaxEnt model with optimized settings, that is (RM = 0.5 and FC = LQ), an AUC value of 0.940 was obtained, far exceeding the accuracy evaluation threshold of 0.9 (Zhang, Jiang, et al. 2024), indicating that the MaxEnt model predicting the distribution of *W. indica* is highly reliable. Although AUC > 0.95 is reported in some studies (Jiang et al. 2025; Xiang et al. 2025), our 0.940 value exceeds the 0.9 excellence threshold (Swets 1988). This reflects robust performance tailored to *W. indica* 's ecology and occurrence data.

### 4.2 Dominant Environmental Factors Affecting the Distribution of *W. indica*

This study identified nine environmental factors affecting the distribution of *W. indica* and their respective contribution rates as follows: mean annual temperature (Bio1, 69.4%), mean diurnal temperature range (Bio2, 12.6%), precipitation of the coldest quarter (Bio19, 9.6%), annual precipitation (Bio12, 3.5%), annual temperature range (Bio7, 2.7%), seasonal precipitation change (Bio15, 0.9%), slope (Slope, 0.7%), maximum temperature of the warmest month (Bio5, 0.5%), and aspect (Aspect, 0.1%). Overall, the cumulative contribution rate of moisture factors to the distribution of *W. indica* is 14%, and that of topographical factors is 0.8%, both of which are significantly lower than the 85.2% contribution rate of temperature factors. Among them, the cumulative contribution of mean annual temperature and mean diurnal temperature range is 82%, making them the primary factors influencing the distribution of *W. indica* in China.

*W. indica* , being a heliophyte, prefers warm and humid climatic conditions, and its growth and reproduction are highly dependent on temperature (Ren et al. 2022). This physiological requirement predisposes *W. indica* to be distributed in warmer regions. Previous studies have suggested that *W. indica* is suitable for growth in areas with an annual average temperature of 16°C–26°C (Ren et al. 2022), whereas this study used the MaxEnt model to predict the potential distribution of *W. indica* and found that its suitable growth annual average temperature range is from −18.9°C to 21.3°C. The discrepancy between the two may stem from various factors, including but not limited to differences in datasets, model algorithms, and the diversity of environmental variable selection (Chen et al. 2012; Zhu et al. 2013). This study considered a broader range of environmental conditions, potentially revealing the potential breadth of temperature adaptability of *W. indica* .

### 4.3 Effects of Climate Change on Potential Distribution of *W. indica*

In this study, a series of significant findings were derived regarding the impact of climate change under current climatic conditions and various future greenhouse gas emission scenarios on the potential suitable habitat of *W. indica* in China: Under current climatic conditions, the potential total suitable habitat area of *W. indica* was found to be 153.31 × 10^4^ km^2^, accounting for 15.97% of China's land area. The low, moderate, and high suitability areas were found to account for 8.17%, 4.37%, and 3.43% of China's land area, respectively, mainly distributed in the southwestern, southeastern, and southern coastal regions of China. Under the SSP1‐2.6 scenario, the total suitable habitat area in the 2050s is projected to be 191.30 × 10^4^ km^2^, and by the 2090s, the total suitable habitat area is slightly increased to 202.42 × 10^4^ km^2^. Under the SSP3‐7.0 and SSP5‐8.5 scenarios, the total suitable habitat area in the 2050s is projected to be 203.22 × 10^4^ km^2^ and 210.19 × 10^4^ km^2^, respectively, with a further increase in the total suitable habitat area by the 2090s. The results of the distribution area changes confirm that the centroid of the potential suitable distribution area of *W. indica* shows a trend of migration towards the northwest, with the migration distance increasing over time. Consistent with the projections by Xu, Miao, et al. (2023), the centroid of the suitable distribution area for apple trees ( *Malus pumila* Mill) is anticipated to move in a north‐northwesterly direction under future climate change scenarios. This shift is likely driven by the need for species to adjust their ranges to higher elevations to maintain optimal growth and reproductive conditions as the climate warms (Zu et al. 2021).

Similar to existing studies (Khan et al. 2022; Liu et al. 2024), the research findings on the potential suitable habitat distribution of *W. indica* in China reveal the significant impact of climate change on species distribution (Román‐Palacios and Wiens 2020). Under scenarios with lower greenhouse gas emissions, the suitable habitat area and range of *W. indica* gradually expand, and under scenarios with higher emissions, this expansion is even more pronounced. This suggests that *W. indica* may have a certain adaptability to climate change, being able to survive under a broader range of climatic conditions. The trend of centroid migration indicates that the distribution of *W. indica* may be undergoing geographical adjustments in response to climate change. This shift may be a direct response to rising temperatures and changing precipitation patterns, as these factors significantly affect plant growth and reproduction (Rauschendorfer et al. 2022). Additionally, the migration of the centroid may also be related to habitat fragmentation and changes in ecosystem services, which could impact the connectivity and survival strategies of species (Echeverría et al. 2007). These findings highlight the potential complex impacts of climate change on biodiversity and ecosystem functions (Harrison et al. 2024; Weiskopf et al. 2020), indicating that these dynamic changes need to be considered when formulating species conservation strategies.

The analysis of the northwestward shift in the geographic distribution centroid of *W. indica* is a critical supplement to simply measuring changes in habitat area. It provides a directional indicator of range dynamics under climate change, offering practical insights for conservation. Specifically, the migration path helps identify potential future dispersal corridors and priority areas for monitoring and assisted migration (Jiang et al. 2025; Xiang et al. 2025), particularly at the leading edge of the range shift. This quantitative, spatially explicit metric translates model predictions into actionable intelligence for designing resilient conservation networks and planning the sustainable management of this valuable medicinal resource in a warming climate (Xia et al. 2022; Yang, Zhu, et al. 2024).

### 4.4 Limitations of Study and Future Research Directions

Our study, while providing critical insights, is subject to several main limitations. First, the MaxEnt model assumes static species‐climate relationships, which may not accurately capture dynamic ecological feedback processes under long‐term climate change. Second, the modeling framework did not incorporate biotic interactions and critical abiotic factors such as soil properties, microclimatic variability, and anthropogenic pressures, all of which collectively influence habitat suitability. Third, reliance on macro‐climatic data may overlook microclimatic heterogeneity, particularly in complex terrains, thereby affecting the precision of local‐scale distribution predictions. Fourth, this study relied solely on the WorldClim climate database; while ensuring global consistency, this approach may not fully capture China's complex local climatic heterogeneity, potentially affecting the fine‐scale precision of habitat predictions. Fifth, the future projections were based on a single climate model (BCC‐CSM2‐MR), which may not fully represent the uncertainty range of future climate scenarios.

Future research should focus on the following directions. First, priority should be given to developing dynamic models that incorporate species dispersal ability and phenotypic plasticity to better simulate dynamic range shifts. Second, subsequent studies need to integrate key variables such as biotic interactions and high‐resolution soil and land‐use data to enhance the practical applicability of predictions for conservation. Third, utilizing satellite‐derived microclimate data and conducting field validations across diverse topographic settings is essential for improving the spatial accuracy of habitat suitability models. Fourth, future studies should incorporate high‐resolution, China‐specific climate datasets (e.g., CN05.1 meteorological data, China Meteorological Forcing Data) to better represent regional topographic and microclimatic variations. Fifth, employing multi‐model ensemble approaches using various CMIP6 GCMs would provide a more robust assessment of distribution trends under climate change.

## 5 Conclusion

This study utilized an optimized MaxEnt model to project the potential distribution of the medicinal plant *W. indica* in China under current and future climate scenarios. The key findings deliver two critical messages for the conservation and utilization of this species. First, our analysis provides quantitative evidence that climate change will significantly reshape the geographic distribution of *W. indica* . The primary evidence is threefold: (1) a substantial expansion of total suitable habitat area by 32.0%–49.6% by the 2090s, (2) a pronounced northwestward shift of the distribution centroid by 76.68–119.91 km, and (3) the identification of annual mean temperature as the dominant controlling factor (69.4% contribution). These model outputs, derived from 902 occurrence points and rigorous CMIP6 scenarios, constitute direct predictive evidence of climate change impacts. Second, the core message is that *W. indica* exhibits significant resilience and a capacity to exploit new suitable habitats under climate change. This suggests potential for increased natural availability but also necessitates proactive strategies. The findings provide a scientific basis for guiding future conservation efforts, such as prioritizing areas for monitoring and designing protected area networks along the northwestern migration corridor, and for planning the sustainable management of this valuable medicinal resource. Future studies should integrate field validation and soil‐microclimatic data to refine these projections.

## Author Contributions

**Yangzhou Xiang:** conceptualization (lead), data curation (lead), formal analysis (lead), funding acquisition (lead), methodology (lead), project administration (lead), validation (lead), visualization (lead), writing – original draft (lead). **Suhang Li:** conceptualization (equal), data curation (lead), formal analysis (lead), methodology (lead), software (lead), validation (lead), visualization (lead), writing – review and editing (lead). **Ying Liu:** conceptualization (lead), methodology (equal), writing – review and editing (equal). **Qiong Yang:** data curation (equal), formal analysis (equal), investigation (equal), methodology (equal), resources (lead), validation (equal), visualization (equal), writing – review and editing (equal). **Jiaxin Yao:** data curation (equal), methodology (equal), resources (equal), software (equal), validation (equal), writing – review and editing (equal). **Huilin Dong:** funding acquisition (lead), investigation (lead), project administration (lead), writing – review and editing (lead). **Bin Yao:** conceptualization (equal), funding acquisition (equal), methodology (equal), project administration (equal), supervision (equal), writing – review and editing (equal). **Yuan Li:** conceptualization (lead), methodology (lead), visualization (equal), writing – original draft (equal), writing – review and editing (lead).

## Conflicts of Interest

The authors declare no conflicts of interest.

## Data Availability

Data Availability Statement: Location records and environmental variables have been uploaded to an open data repository via Figshare (https://doi.org/10.6084/m9.figshare.29429027.v1).
